# Neutrophil-to-Lymphocyte Ratio in Acute Exacerbation of Idiopathic Pulmonary Fibrosis

**DOI:** 10.3390/jcm12237446

**Published:** 2023-11-30

**Authors:** Toru Arai, Takayuki Takimoto, Naoko Takeuchi, Shojiro Minomo, Tomoko Kagawa, Yoshikazu Inoue

**Affiliations:** 1 Clinical Research Center, National Hospital Organization Kinki-Chuo Chest Medical Center, Sakai City 591-8555, Japan; takimoto.takayuki.ra@mail.hosp.go.jp (T.T.); giichiyi@me.com (Y.I.); 2 Department of Respiratory Medicine, National Hospital Organization Kinki-Chuo Chest Medical Center, Sakai City 591-8555, Japan; takeuchi.naoko.hb@mail.hosp.go.jp (N.T.); minomo.shojiro.mh@mail.hosp.go.jp (S.M.); kagawa.tomoko.qw@mail.hosp.go.jp (T.K.)

**Keywords:** neutrophil-to-lymphocyte ratio, idiopathic pulmonary fibrosis, acute exacerbation, prognosis

## Abstract

Background: This study aimed to clarify the prognostic value of the neutrophil-to-lymphocyte ratio (NLR) in patients with acute exacerbation of idiopathic pulmonary fibrosis (AE-IPF). Methods: Eighty-six patients diagnosed with AE-IPF were included in this single-center retrospective study. The NLR was calculated by dividing the peripheral neutrophil count by the peripheral lymphocyte count. The cut-off values of the NLR for predicting 90-day survival were determined using receiver operating characteristic curve analysis. Oxygenation deterioration on days 4 and 8 relative to that on day 1 was clinically defined. The prognostic value of NLR was evaluated using Cox proportional hazard regression analysis. Results: The cut-off value of day-1, day-4, and day-8 NLRs for predicting 90-day survival was 12.13, 14.90, and 10.56, respectively. A higher day-1 NLR was a significant predictor of a poor prognosis in univariate and multivariate analyses. Survival was significantly better in patients without oxygenation deterioration on days 4 and 8 than in those with deterioration. Day-4 and day-8 NLR could predict 90-day survival in patients without oxygenation deterioration. Conclusions: Day-1 NLR was a useful predictor of 90-day survival in AE-IPF. Further, monitoring day-4 and day-8 NLRs and evaluating oxygenation deterioration may be useful for managing AE-IPF.

## 1. Introduction

Idiopathic pulmonary fibrosis (IPF) is a lung disease of unknown etiology with a poor prognosis [1,2]. Some patients with IPF experience rapid deterioration resulting in unpredictable death [1,2,3,4]; such cases of acute deterioration with unknown etiology are known as acute exacerbation (AE) of IPF (AE-IPF). Although treatment with corticosteroids is usually performed, in accordance with the 2011 American Thoracic Society (ATS)/European Respiratory Society (ERS)/Japanese Respiratory Society (JRS)/Latin American Thoracic Association (ALAT) guidelines for the diagnosis and management of IPF [1], AE is the most common cause of death in IPF [4].

AE-IPF severity at its onset is usually evaluated by the ratio of arterial oxygen tension (PaO_2_)/fraction of inspired oxygen (FiO_2_) (PaO_2_/FiO_2_) [5,6,7]. Serum levels of Krebs von den Lungen (KL)-6 are useful in predicting survival in AE-IPF [7,8]. However, timely re-evaluation with fewer burdensome parameters is important for managing AE-IPF. The neutrophil-to-lymphocyte ratio (NLR) is an easily available biomarker, calculated using white blood cell counts. In 2001, Zahorec et al. first proposed the NLR as a severity marker of infection in clinical practice [9]. The NLR represents the balance between inflammatory activator neutrophils and inflammatory regulator lymphocytes; the higher the value, the more unbalanced the inflammatory state [10]. Hence, it is useful for predicting the prognosis in acute respiratory distress syndrome (ARDS) [11], sepsis [12], pancreatitis [10], and coronavirus disease 2019 [13]. The pathophysiological similarity between ARDS and AE-IPF suggests a prognostic value of NLR in AE-IPF; however, this has not been investigated. Therefore, this study aimed to clarify the value of the NLR for predicting survival in AE-IPF.

## 2. Materials and Methods

### 2.1. Subjects

From the inpatients database of interstitial lung diseases, we retrospectively identified 113 patients with AE of idiopathic interstitial pneumonia (IIP) treated at the Kinki-Chuo Chest Medical Center between January 2004 and February 2016. The underlying IIP was re-evaluated according to updated 2022 ATS/ERS/JRS/ALAT guidelines for IPF [14]. Patients with IIP with a probable usual interstitial pneumonia pattern on high-resolution computed tomography (HRCT) before AE were diagnosed with clinical IPF after a clinico-radiological discussion. Chronic hypersensitivity pneumonia was ruled out. Further, our cohort did not include patients with collagen vascular disease-associated interstitial lung disease diagnosed using specific diagnostic criteria. Finally, 86 patients with AE-IPF were enrolled in this study, comprising 27 with histologically diagnosed IPF and 59 clinical IPF cases. Serum autoantibody positivity was evaluated based on the criterion of “interstitial pneumonia with autoimmune features (IPAF)” in the serological domain and anti-neutrophil cytoplasmic antibody levels [15].

Our institutional review board approved this study (Rin-2022-047, acceptance date: 10 August 2022). Because of its retrospective nature, the requirement for informed consent was waived.

### 2.2. Diagnosis of AE-IPF

AE-IPF was diagnosed according to ATS diagnostic criteria [4]. Triggered AE cases were included. An acute (typically defined as <1 month in duration), clinically significant deterioration in IPF was characterized by newly appeared ground glass opacity/consolidation that could not be fully explained by cardiac failure or volume overload. The infectious etiology was investigated by measuring antibodies for Mycoplasma pneumoniae and Chlamydia pneumoniae in paired sera, β-D glucan, cytomegalovirus antigen, and bacterial cultures of blood and sputum. Endotracheal aspiration or bronchoalveolar lavage was performed in some cases [16].

### 2.3. HRCT Findings at AE-IPF Diagnosis

The HRCT pattern at AE onset was classified by chest radiologists into one of three patterns: peripheral, multifocal, or diffuse [3]. The HRCT pattern was further classified as diffuse or non-diffuse.

### 2.4. AE-IPF Treatment

AE-IPF is generally treated with prednisolone following intravenous methylprednisolone (500–1000 mg/day for 3 successive days), with or without an immunosuppressant [16,17]. In 20 patients, we performed polymyxin B-immobilized fiber column direct hemoperfusion therapy [17] using Toraymyxin^®^ (Toray Medical, Tokyo, Japan). In 8 patients, intravenous soluble thrombomodulin (380 U/kg/day for 6 days) was administered [18]. Positive-pressure ventilation (PPV), including non-invasive PPV (*n* = 20) and/or invasive PPV (*n* = 14), and nasal high-flow therapy were performed to maintain oxygenation.

### 2.5. Collection of Clinical Data

Clinical data, including blood test results and AE-IPF management and outcomes, were collected from medical records. Serum KL-6 levels were measured using a commercial ELISA kit (Eisai, Tokyo, Japan), with a cut-off level of 500 U/mL [19]. The PaO_2_/FiO_2_ ratio was classified into ≤200/>200 [20].

### 2.6. NLR Calculation

The NLRs (day-1, day-4, and day-8 NLRs) were calculated by dividing the peripheral neutrophil count by the peripheral lymphocyte count at the onset of AE-IPF and at 3 and 7 days after AE onset, that is, on days 1, 4, and 8, respectively.

### 2.7. Oxygenation Deterioration on Days 4 and 8

Oxygenation deterioration was defined according to modified criteria reported by Blancal et al. [21], as satisfying at least one of the following criteria: decrease in PaO_2_ or increase in alveolar arterial oxygen difference AaDO_2_ ≥ 10 Torr; decrease in percutaneous oxygen saturation SpO_2_ ≥ 5%; increase in supplemental oxygen ≥ 3 L/min; or a step up in oxygen inhalation device in the following order: nasal cannula, oxymizer, mask, reservoir mask, high-flow nasal oxygen, non-invasive PPV, and invasive PPV.

### 2.8. Statistical Analysis

Continuous variables were presented as medians with interquartile range (IQR). A normality check was performed using Shapiro–Wilk test and Kolmogorov–Smirnov test. Correlations between the NLR and other parameters were evaluated using the Wilcoxon rank-sum test or Spearman’s rank correlation analysis. Survival was observed from day 1 to day 91, and Kaplan–Meier analysis with log-rank tests were used to evaluate the 90-day survival. Day-4 and day-8 survivors refer to 3-day and 7-day survivors who were alive on day 4 and day 8, respectively. Univariate Cox proportional hazards regression analysis was used to calculate hazard ratios (HRs) for 90-day mortality. Prognostic factors were determined using multivariate analysis with a stepwise selection procedure. The prognostic value of oxygenation deterioration and the NLR on days 4 and 8 was also evaluated in day-4 and day-8 survivors, respectively. The cut-off values of the day-1 NLR for predicting 90-day mortality were examined using receiver operating characteristic (ROC) curve analysis. Additionally, the cut-off levels of the day-4 and day-8 NLR for predicting 90-day mortality among day-4 and day-8 survivors, respectively, were examined using ROC curve analysis. The sensitivity of higher day-1, day-4, and day-8 NLRs was calculated by dividing the number of 90-day dead patients with each NLR > cut-off levels by that of all 90-day dead patients who received each NLR evaluation. Specificity was calculated by dividing number of 90-day alive patients with each NLR ≤ cut-off levels by that of all 90-day alive patients who received each NLR evaluation. Statistical significance was set at *p* < 0.05. All statistical analyses were performed using SPSS version 26 for Macintosh (IBM Corp., Armonk, New York, NY, USA).

## 3. Results

### 3.1. Patient Demographics

Patient demographics are presented in Table 1. The median age at AE-IPF diagnosis was 72 years, and 72 patients were male. Long-term oxygen therapy was administered to 28 patients. Pirfenidone and nintedanib were administered at the time of AE-IPF diagnosis in 4 and 1 patient, respectively.

### 3.2. Outcomes

**Figure 1:**
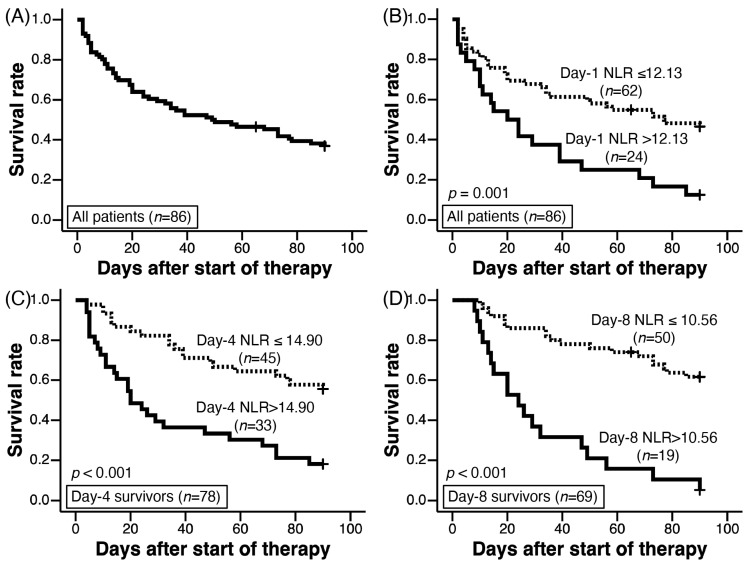
Kaplan–Meier analysis with log-rank tests were used to evaluate the 90-day survival. (**A**) showed 90-day survival of all patients (*n* = 86). Among all patients (*n* = 86), 90-day survival of patients with day-1 NLR > 12.13 (solid line) was worse than those with day-1 NLR ≤ 12.13 (dotted line) on day-1 (log-rank test, *p* < 0.001, (**B**)). Among day-4 (**C**) and day-8 survivors (**D**), 90-day survival of patients with day-4 and day-8 NLRs > cut-off values were worse than those with NLRs ≤ cut-off levels, respectively. Cut-off values of day-4 and day-8 NLRs were 14.90 and 10.56, respectively.

There were 79 and 71 day-4 and day-8 survivors, respectively. The 90-day survival rate was 36.5% (31/85 patients); one patient was censored on day 66. The overall median survival time was 49 days (Figure 1A). Oxygenation deterioration on day 4 occurred in 24/79 day-4 survivors (30.3%), and that on day 8 occurred in 19/71 day-8 survivors (26.7%).

### 3.3. Peripheral Blood Findings

The median (IQR) NLR of survivors on days 1 (*n* = 86), 4 (*n* = 78), and 8 (*n* = 69) was 7.56 (4.61–12.90, Table 1), 12.10 (7.17–21.26, Table 2), and 5.20 (3.47–11.14), respectively. The day-4 NLR was significantly higher than the day-1 and day-8 NLR (Wilcoxon signed-rank test, *p* < 0.001) (Table 2).

### 3.4. Oxygen Deterioration on Days 4 and 8

**Figure 2:**
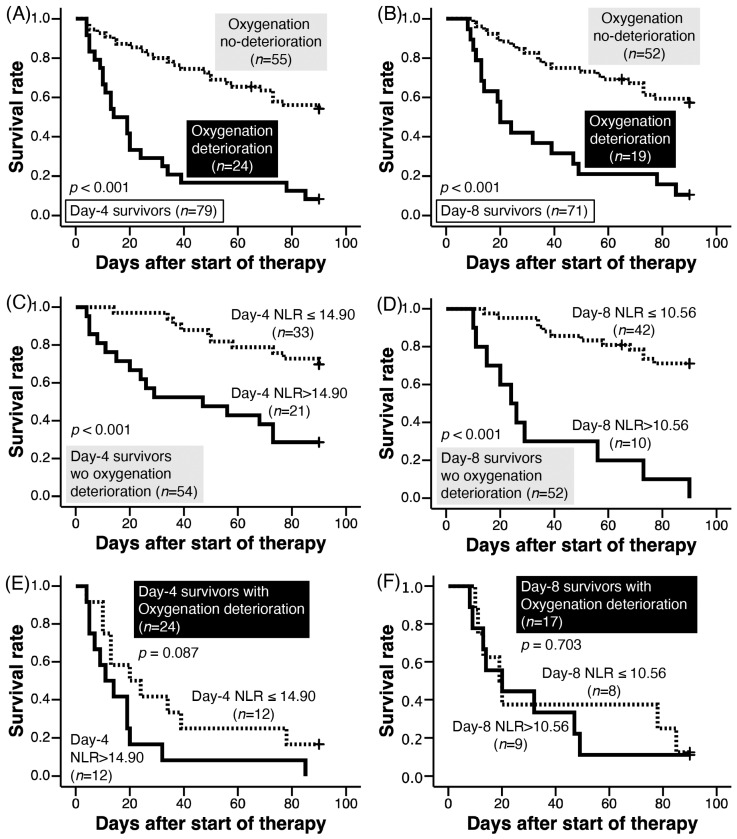
Among day-4 survivors (*n* = 79) (**A**) and day-8 survivors (*n* = 72) (**B**), 90-day survival of patients with oxygenation deteriorations (solid line) was worse than that of patients without oxygenation deteriorations (dotted line) (log rank test, *p* < 0.001). For day-4 survivors without oxygenation deterioration on day 4 ((**C**); *n* = 54; NLR was not evaluated in 1 of 55 patients shown in (**A**)) and day-8 survivors without oxygenation deterioration on day 8 ((**D**); *n* = 52), higher NLR (solid line) suggested poorer 90-day survival compared to those with lower NLR (dotted line) (log rank test; *p* = 0.001 and *p* < 0.001, respectively). For day-4 survivors with oxygenation deterioration on day 4 ((**E**); *n* = 24) and day-8 survivors with oxygen deterioration on day 8 ((**F**); *n* = 17; NLR was not evaluated in 2 of 19 patients shown in (**A**)), there was no significant difference in 90-day survival between patients with higher NLR (solid line) and lower NLR (dotted line) (log rank test; *p* = 0.087 and *p* = 0.703, respectively). Cut-off levels of the day-4 and day-8 NLRs to determine higher and lower NLRs were 14.90 (**C**,**E**) and 10.56 (**D**,**F**), respectively. Abbreviations: NLR, neutrophil-to-lymphocyte ratio.

Ninety-day survival was significantly worse in patients with oxygenation deterioration on days 4 (Figure 2A) and 8 (Figure 2B) than in patients without oxygenation deterioration (log-rank test, *p* < 0.001). On day 4, lactate dehydrogenase (LDH) levels were significantly higher in patients with oxygen deterioration than in patients without oxygen deterioration although day-4 NLR were not (Table 3). On day 8, the NLR, LDH, and C-reactive protein (CRP) levels were significantly higher in patients with oxygenation deterioration than in patients without oxygenation deterioration (Table 3).

### 3.5. Correlations between the NLR and Other Parameters on Day 1

The day-1 NLR was significantly correlated with age (Rho 0.288, *p* = 0.007), PaO_2_/FiO_2_ ratio (Rho 0.231, *p* = 0.033), white blood cell count (Rho 0.419, *p* < 0.001), neutrophil count (Rho 0.636, *p* < 0.001), lymphocyte count (Rho −0.785, *p* < 0.001), and HRCT pattern (Rho 0.332, *p* = 0.002) (Spearman’s rank correlation, Table 4). In addition, the day-1 NLR was significantly larger in patients with a diffuse HRCT pattern (median 11.94, IQR 6.93–18.53) than in patients with a non-diffuse pattern (median 6.48, IQR 3.64–11.35) (Mann–Whitney U test, *p* = 0.002).

### 3.6. Cut-Off Values of NLR on Day 1, Day 4, and Day 8 for Predicting 90-Day Survival

The cut-off value of the day-1 NLR for predicting 90-day survival in 85 patients (one censored patient was excluded) was 12.13 (AUC 0.712, 95% CI 0.602–0.823, *p* = 0.001, sensitivity 38.9%, specificity 90.3%). Among all patients (*n* = 86) with AE-IPF, the 90-day survival of patients with NLR > 12.13 (*n* = 24) was significantly worse than that of patients with NLR ≤ 12.13 (*n* = 62) (log rank test, *p* = 0.001, Figure 1B). The cut-off values of the day-4 and day-8 NLR for predicting 90-day survival from day 4 and day 8 were 14.90 (AUC 0.684, 95% CI 0.567–0.801, *p* = 0.006, sensitivity 57.4%, specificity 80.6%) and 10.56 (AUC 0.718, 95% CI 0.666–0.883, *p* < 0.001, sensitivity 48.6%, specificity 96.8%), respectively (Table 5). For day-4 survivors and day-8 survivors, higher day-4 and day-8 NLRs more than each cut-off value show worse 90-day survival by Kaplan–Meier analysis (Figure 1C and Figure 1D, *p* < 0.001 and *p* < 0.001, respectively).

### 3.7. Prognostic Factors of AE-IPF on Day 1 Examined by Cox Proportional Hazard Regression Analysis

**Figure 3:**
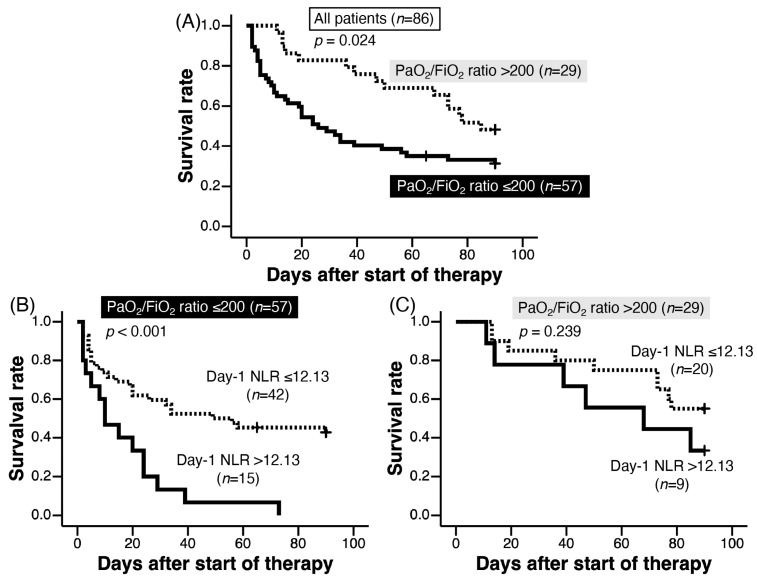
Among all patients (*n* = 86, (**A**)), 90-day survival of patients with PaO_2_/FiO_2_ ratio > 200 was significantly better than those with PaO_2_/FiO_2_ ratio ≤ 200 (log-rank test, *p* = 0.024). For patients with PaO_2_/FiO_2_ ratio > 200 (*n* = 57, (**B**)), day-1 NLR > 12.13 (solid line) suggested poorer 90-day survival compared to day-1 NLR ≤ 12.13 (dotted line) (log-rank test, *p* < 0.001). However, for patients with PaO_2_/FiO_2_ ratio ≤ 200 (*n* = 29, (**C**)), there was no significant difference in 90-day survival between day-1 NLR ≤ 12.13 (dotted line) and day-1 NLR > 12.13 (solid line) (log-rank test, *p* = 0.239).

Cox proportional hazard regression analysis results are shown in Table 6. Univariate analysis revealed that a PaO_2_/FiO_2_ ratio ≤ 200, lower lymphocyte count, higher day-1 NLR, and diffuse HRCT pattern were significant predictors of a poor prognosis. Multivariate analysis with a stepwise selection procedure revealed PaO_2_/FiO_2_ ratio ≤ 200 and higher day-1 NLR as significant predictors of a poor prognosis. A day-1 NLR more than each cut-off value was also a significant predictor after the adjustment with a PaO_2_/FiO_2_ ratio ≤ 200 (Table 6). Kaplan–Meier analysis showed that the survival of patients with PaO_2_/FiO_2_ ratio ≤ 200 was worse than that with a PaO_2_/FiO_2_ ratio > 200 (log-rank test, *p* = 0.024, Figure 3A). A day-1 NLR > 12.13 revealed significantly higher mortality for patients with PaO_2_/FiO_2_ ratio ≤ 200 (log-rank test, *p* < 0.001, Figure 3B); however, it was not associated with mortality for patients with a PaO_2_/FiO_2_ ratio > 200 (log-rank test, *p* = 0.239, Figure 3C).

### 3.8. Prognostic Factors of AE-IPF Survivors on Day 4 and Day 8 Examined by Cox Proportional Hazard Regression Analysis

Univariate analysis revealed that a day-4 NLR and day-4 NLR more than the cut-off value was a significant prognostic factor for day-4 survivors (Table 7). Multivariate analysis with stepwise procedure showed these two factors were significant prognostic factors, similar to LDH and oxygenation deterioration on day 4. A day-8 NLR and day-8 NLR more than the cut-off value were significant prognostic factors for day-8 survivors by univariate and multivariate analysis, similarly for day-4 survivors. Oxygenation deterioration on day-8 also reflected 90-day mortality similarly to the two day-8 NLR parameters (Table 7).

### 3.9. NLR and Survival of Patients without Oxygenation Deterioration on Days 4 and 8

Among patients without oxygenation deterioration on day 4, survival was better in those with a lower NLR than in those with a higher NLR (Figure 2C); a similar association between survival and NLR was also observed on day 8 (Figure 2D). Among patients without oxygenation deterioration, treatment for AE-IPF, including the dose of corticosteroids and frequency of immunosuppressants administration, was not significantly different between those with high and low NLRs on days 4 and 8 (Table 8).

### 3.10. NLR and Survival of Patients with Oxygenation Deterioration on Days 4 and 8

Among patients with oxygenation deterioration on day 4, survival was similar between those with lower NLR and higher NLR (Figure 2E); a similar association between survival and NLR was also observed on day 8 (Figure 2F).

## 4. Discussion

In this study, we demonstrated the importance of the NLR at the onset of AE-IPF for predicting 90-day survival. Patients with AE-IPF without oxygenation deterioration on days 4 and 8 survived significantly longer than those with oxygenation deterioration. The day-4 and day-8 NLR was associated with 90-day survival in patients without oxygenation deterioration on day 4 and day 8, respectively, but not in those with oxygenation deterioration.

As a biomarker of AE-IPF, KL-6 has been reported to reflect survival in AE-IPF [7]. We have also reported that the combination of HRCT pattern and change in serum KL-6 level from a stable state is useful for predicting survival in patients with AE-IIP [22]. Kishaba et al. proposed staging systems for AE-IPF, including serum KL-6 levels [7]. However, KL-6 levels can only be measured in a limited number of countries. Additionally, it is difficult to perform frequent measurements for AE-IPF management adjustment. The NLR is easily and frequently evaluated worldwide, and its advantage over KL-6 cannot be overemphasized. The NLR can be calculated from neutrophil and lymphocyte counts in the peripheral blood, and understanding the changes in these cell counts is important.

Neutrophils are the first immune cells recruited to the site of inflammation following stimulation by chemotactic factors released from damaged pulmonary tissues [23]. First, the production of cytokines triggers the release of immature granulocytes from the bone marrow pool, indicated by the presence of immature cells in peripheral circulation [24]. The neutrophil maturation time in the bone marrow is reduced from 6–8 days (basal condition) to 3–4 days during active neutrophil recruitment [25]. Bone marrow neutrophil production is also increased by an acute inflammatory state [25]. Based on these mechanisms, neutrophilia occurs in patients with pneumonia and ARDS, similar to that in AE-IPF.

Lymphopenia has emerged as a prominent feature in patients with sepsis and is associated with a poor prognosis. Lymphocyte death has been observed in Gram-negative bacteria-derived lipopolysaccharide-induced injury in cellular and acute animal models [26]. Accurate mechanisms underlying lymphopenia in sepsis are still lacking; however, the massive migration of lymphocytes to the lungs, adhesion to the vascular endothelium, impaired production in the bone marrow, and increases in apoptotic pathways during the acute phase of pneumonia may contribute to lymphopenia [27]. Lymphopenia is associated with a poor prognosis in ARDS [27] and is thought to have pathophysiological similarities to AE-IPF. We also firstly suggested that, in patients with AE-IPF, lower day-1 lymphocyte counts are a significant predictor of a poor prognosis by univariate analysis.

The pathophysiological aspects of AE-IPF are similar to those of ARDS. NLR has been reported as a useful prognostic factor in ARDS. Wang et al. reported an NLR cut-off value for ARDS of 14.0 [28]. A higher NLR has also been reported as being associated with a poor prognosis in IPF [29,30]. Chen et al. reported that the NLR was significantly higher in patients with AE-IPF than in patients with stable IPF, indicating that it as a potential predictor of the prognosis in AE-IPF; however, the authors did not examine its prognostic value [30]. In the present study, the cut-off value of the day-1 NLR for the diagnosis of AE was 12.13; additionally, a higher NLR was a significant predictor of poor survival after adjusting for other clinical parameters.

Diffuse, multifocal, and peripheral HRCT patterns have been reported as significant predictors of survival in AE-IPF. AE-IPF with a diffuse HRCT pattern suggests pathological diffuse alveolar damage [3]. The day-1 NLR was significantly higher in patients with a diffuse pattern than in those with a non-diffuse pattern. HRCT findings are important for diagnosing AE-IPF, and HRCT patterns are important for predicting survival; however, in severe cases, HRCT pattern evaluation might be impossible. Hence, the NLR can be used as a surrogate marker for the HRCT pattern, suggesting the pathophysiology and prognosis of patients with AE-IPF, especially for those who cannot undergo HRCT.

The day-1 NLR was a significant prognostic factor in AE-IPF, similar to that for ARDS. The day-1 NLR was significantly associated with the severity of AE-IPF, as suggested by the PaO_2_/FiO_2_ ratio; however, the day-4 and day-8 NLR may have been influenced not only by AE-IPF severity and pathophysiology, but also by corticosteroid administration. Generally, corticosteroid administration induces neutrophilia and lymphopenia [31,32]. However, we showed that corticosteroid therapy in AE-IPF induced a significant increase in day-8 lymphocyte counts in patients without oxygenation deterioration. As shown previously, steroids directly induce lymphocyte apoptosis, while simultaneously inhibiting the production of inflammatory cytokines [33], which induce the activation-induced cell death of lymphocytes and endothelial adhesion molecules for lymphocytes. Hence, steroids may have induced a temporary increase in lymphocyte counts, suggesting the sufficient anti-inflammatory effects of steroids on day 8. This lymphocyte count recovery on day 8 led to a decrease in the NLR on day 8 and suggested a good prognosis in patients without oxygenation deterioration (Table 4). If the day-8 NLR is increased relative to the day-1 NLR, immunosuppression might be insufficient to suppress pulmonary inflammation; accordingly secondary steroid pulse therapy or additional immunosuppressants might be needed. We did not find any significant difference in AE treatment between patients with high and low NLR among those without oxygenation deterioration (Table 8). Cases with a poor prognosis can be identified using the day-4 and day-8 NLRs in patients without oxygenation deterioration, and measures should be taken to improve the prognosis in such patients.

The selection of additional candidate therapies to improve the prognosis of AE-IPF is an important problem. Considering that the NLR can predict a poor prognosis in AE-IPF, anti-inflammatory drugs may be candidates. Intravenous cyclophosphamide pulse therapy is administered for AE-IPF to achieve rapid immunosuppressive effects [34]. Small-scale studies using historical controls have shown promising effects of thrombomodulin on AE-IPF [18,35] through anti-inflammatory effects [36]. In contrast, previous randomized trials have reported that both drugs are harmful in AE-IPF [37,38]; however, if these drugs are only administered to patients with true necessity based on oxygenation deterioration and their NLR, they might show some beneficial effects in AE-IPF. A new phosphodiesterase 4 B inhibitor, BI 1015550, which is under phase 3 trials for IPF, might be a candidate [39] because it has been shown to inhibit tumor necrosis factor-α and interleukin-2 release from peripheral mononuclear cells in vitro and lipopolysaccharide-induced neutrophil influx into the bronchoalveolar lavage in an in vivo rat model [40].

This study had some limitations, including its retrospective, single-center design. Additionally, oxygenation deterioration on days 4 and 8 was not evaluated using arterial blood gas analyses. However, we used the criteria for deteriorated oxygenation described in a previous report [21], which we consider as useful in real-world clinical settings. Further, limited parameters, including CRP and LDH levels, in addition to the NLR, were examined as prognostic factors on days 4 and 8; however, novel parameters were beyond the scope of this study, which aimed to find easily re-evaluable parameters that can be measured in most countries. Other inflammatory systemic biomarkers, including the monocyte-to-lymphocyte ratio, platelet-to-lymphocyte ratio, and systemic immune-inflammation index, were not examined, and additional future studies are needed to determine most important parameters [41].

## 5. Conclusions

The day-1 NLR is a useful predictor of 90-day survival in patients with AE-IPF. Further, monitoring the day-4 and day-8 NLRs and evaluating oxygenation deterioration may be useful for managing AE-IPF.

## Figures and Tables

**Table 1:** Demographics of patients with AE-IPF.

Parameters	Total Cases (*n* = 86)
**Before AE**	
Age, years	72 (66.0–75.25)
Sex, male/female	72/14
Smoking, NS/CS or Ex	18/68
IPF, histologically diagnosed/clinical	27 */59
Autoantibody, yes/no	14/72
PSL before AE, yes/no	20/66
Immunosuppressants, yes/no	14/72
Initial immunosuppressants, AZP/CyA/CPA	9/3/2
Pirfenidone, yes/no	4/82
Nintedanib, yes/no	1/85
LTOT, yes/no	28/58
**At the onset of AE (day 1)**	
Triggered, yes/no	11 **/75
PaO_2_/FiO_2_ ratio, Torr	155.4 (85.0–227.0)
PaO_2_/FiO_2_ ratio, ≤200/>200	57/29
KL-6, ×100 U/mL	15.40 (9.24–21.62)
WBC, ×10^9^/L	10.15 (8.10–13.22)
Neutrophils, ×10^9^/L	8.61 (6.08–11.60)
Lymphocytes, ×10^9^/L	1.10 (0.70–1.60)
NLR	7.56 (4.61–12.90)
CRP, mg/dL	11.57 (4.55–16.80)
LDH, U/L	355.0 (296.5–423.5)
HRCT, diffuse/non-diffuse	26/60
HRCT, diffuse/multifocal/peripheral	26/40/20
**Treatment for AE**	
Intravenous high-dose methylprednisolone, yes/no	86/0
Initial dose of PSL, mg/kg	0.933 (0.796–1.000)
Immunosuppressant, yes/no	43/43
CPA pulse, yes/no	18/68
AZP/CyA	16/12
Recombinant soluble thrombomodulin, yes/no	8/78
PPV within a month from the start of treatment, yes/no	34/52
NPPV/IPPV ^§^	20/14
PMX-DHP therapy ^†^, yes/no	20/66
Pirfenidone, yes/no	3/83
Nintedanib, yes/no	0/86
**Outcomes**	
Day-4 survivors (3-day survivors), yes/no	79/7
Oxygenation deterioration on day 4, yes/no	24/55
Day-8 survivors (7-day survivors), yes/no	71/15
Oxygenation deterioration on day 8, yes/no	19/52
Day-91 survivors (90-day survivors) ^#^, yes/no/unknown	31/54/1
Median survival time, days	49

Abbreviations: AE, acute exacerbation; AZP, azathioprine; CPA, cyclophosphamide; CRP, C-reactive protein; CS, current smoker; CyA, cyclosporine A; EX, ex-smoker; HRCT, high-resolution computed tomography; IPF, idiopathic pulmonary fibrosis; IPPV, invasive positive pressure ventilation; KL-6, Krebs von den Lungen-6; LDH, lactate dehydrogenase; LTOT, long-term oxygen therapy; NLR, neutrophil-to-lymphocyte ratio; NPPV, non-invasive positive pressure ventilation; NS, non-smoker; PaO_2_/FiO_2_ ratio; PMX-DHP therapy, hemoperfusion therapy with polymyxin B-immobilized fiber column; PPV, positive-pressure ventilation; PSL, prednisolone; WBC, white blood cells. The data are presented as the frequency or median (interquartile range) for each parameter. * Diagnosed by specimens obtained by surgical lung biopsy (*n* = 2) and autopsy (*n* = 25). ** Among patients with AE-IPF, 75 (87.2%) patients were classified as idiopathic AE, and 11 patients (12.8%) were classified as triggered AE (infection, *n* = 2; post-procedure/post-operation, *n* = 5; drug toxicity, *n* = 1; other causes, *n* = 3). ^§^ Cases treated with both NPPV and IPPV were classified as IPPV-treated cases. ^†^ Patients who received PMX-DHP therapy for the second AE were not included as PMX-DHP therapy-treated cases. ^#^ There was one censored case, and 90-day survival was unknown in the case.

**Table 2:** Change of peripheral blood findings from day 1 to day 8 *.

	Neutrophils, ×10^9^/L	Lymphocytes, ×10^9^/μL	NLR
**Day-4 survivors (*n* = 78 ^§^)**			
Day 1	8.61 (6.03–11.62)	1.14 (0.73–1.60)	7.36 (4.53–12.17)
Day 4	10.15 (8.20–14.12)	0.90 (0.50–1.30)	12.10 (7.17–21.26)
*p*-value *			
Day 1 vs. Day 4	<0.001	<0.001	<0.001
**Day-8 survivors (*n* = 68 ^†^)**			
Day 1	8.47 (6.06–11.47)	1.14 (0.75–1.67)	7.12 (4.19–12.15)
Day 4	10.10 (7.60–13.15)	0.90 (0.50–1.33)	11.94 (6.40–18.24)
Day 8	10.03 (7.80–12.00)	1.60 (0.90–2.17)	5.21 (3.48–11.23)
*p*-value *			
Day 1 vs. Day 8	0.003	<0.001	0.521
Day 4 vs. Day 8	0.290	<0.001	<0.001

Abbreviations: NLR, neutrophil-to-lymphocyte ratio. Median (interquartile range) of each parameter was shown. * Peripheral blood findings were compared by Wilcoxon signed-rank test. ^§^ Day-4 NLR was not evaluated in 1 patient, although 78 of 79 patients were alive on day 4. ^†^ Day-8 NLR was not evaluated in 2 patients, and all Day-1, day-4, and day-8 NLRs were evaluated in 68 of 71 patients alive on day 8.

**Table 3:** Parameters on day 4 and day 8 between oxygenation deteriorated and non-deteriorated cases.

Parameters	Deterioration	Non-Deterioration	*p*-Value
On day 4 (*n* = 78 *)	*n* = 24	*n* = 54 **	
Day-4 NLR	15.25 (8.48–24.75)	11.94 (6.48–19.43)	0.443
NLR increase on day 4 from day 1	6.42 (−5.11–14.69)	4.01 (−0.22–8.89)	0.779
NLR increase on day 4 from day 1, >0/≤0	15/9	40/14	0.420
Neutrophils on day 4, ×10^9^/L	10.30 (8.90–13.57)	9.80 (8.05–14.28)	0.753
Lymphocytes on day 4, ×10^9^/L	0.88 (0.45–1.13)	0.90 (0.50–1.36)	0.528
LDH on day 4, U/L	385 (352–510) ^#^	306 (264–375)	<0.001
CRP on day 4, mg/dL	4.36 (2.49–8.88)	2.60 (1.13–7.50)	0.295
**On day 8 (*n* = 69 ^†^)**	*n* = 17 ^‡^	*n* = 52	
Day-8 NLR	10.94 (6.33–21.50)	4.82 (3.25–9.07)	0.002
NLR increase on day 8 from day 1	2.19 (−8.19–13.95)	−0.89 (−3.56–1.93)	0.303
NLR increase on day 8 from day 1, >0/≤0	10/7	20/32	0.167
Neutrophils on day 8, ×10^9^/L	10.36 (8.05–12.60)	9.75 (7.34–12.00)	0.456
Lymphocytes on day 8, ×10^9^/L	0.929 (0.650–1.38)	1.91 (1.20–2.46)	0.001
LDH on day 8, U/L	388 (326–477)	278 (237–355)	<0.001
CRP on day 8, mg/dL	4.61 (1.00–11.14)	1.33 (0.55–3.51)	0.012

Abbreviations: AE, acute exacerbation; CRP, C-reactive protein; LDH, lactate dehydrogenase; NLR, neutrophil-to-lymphocyte ratio. Number or median (interquartile range) of each parameter was shown. *: Seventy-nine cases were alive on day 4; however, no data were available in one case. **: Fifty-five cases were alive on day 4 with no oxygenation deterioration; however, no data were available in one case. ^#^: *n* = 23. ^†^: Seventy-one cases were alive on day 8; however, no data were available in two cases. ^‡^: Nineteen cases were alive on day 8 with oxygenation deterioration; however, no data were available in two cases.

**Table 4:** Correlation between NLR and other parameters (Spearman’s rank correlation).

	Rho	*p*-Value
**Before AE**		
Age, years	0.288	0.007
Sex, male/female	0.199	0.066
Smoking, CS or Ex/NS	−0.079	0.467
SLB or autopsy for underlying IPF, yes/no	0.022	0.839
Autoantibody, yes/no	0.060	0.581
PSL before AE, yes/no	0.208	0.054
LTOT, yes/no	0.076	0.487
**At the onset of AE**		
Triggered, yes/no	−0.090	0.407
PaO_2_/FiO_2_ ratio, Torr	0.231	0.033
PaO_2_/FiO_2_ ratio, ≤200/>200	0.182	0.093
KL-6, ×100 U/mL (*n* = 82)	−0.088	0.433
CRP, mg/dL	0.210	0.053
LDH, U/L	0.176	0.104
HRCT, diffuse/non-diffuse	0.332	0.002

Abbreviations: AE, acute exacerbation; CRP, C-reactive protein; CS, current smoker; EX, ex-smoker; HRCT, high-resolution computed tomography; IPF, idiopathic pulmonary fibrosis; KL-6, Krebs von den Lungen-6; LDH, lactate dehydrogenase; LTOT, long-term oxygen therapy; NLR, neutrophil-to-lymphocyte ratio; NS, non-smoker; P/F ratio, PaO_2_/FiO_2_ ratio; PSL, prednisolone; SLB, surgical lung biopsy.

**Table 5:** Cut-off levels of day-1, day-4, day-8 NLRs to predict 90-day mortality from day 1, day 4, and day 8, respectively.

Parameters	n	Cutoff	AUC	95% CI	*p*-Value	Sensitivity ^§^	Specificity ^#^
Day-1 NLR	85 *	12.13	0.712	0.602–0.823	0.001	38.9%	90.3%
Day-4 NLR	78 **	14.90	0.684	0.567–0.801	0.006	57.4%	80.6%
Day-8 NLR	69 ^¶^	10.56	0.774	0.666–0.883	<0.001	48.6%	96.8%

Abbreviations: AUC, area under the curve; CI, confidence interval; NLR, neutrophil-to-lymphocyte ratio. *: Cut-off levels of day-1 NLR to predict 90-day mortality for all AE-IPF cases except for one censored case (*n* = 85). **: Day-4 survivors were 79 patients, and day-4 NLR was not available in one patient. ^¶^: Day-8 survivors were 71 patients, and day-8 NLR was not available in two patients. ^§^: Sensitivity of day-1, day-4, and day-8 NLRs for predicting 90-day mortality was calculated by dividing number of dead patients on day 91 with each NLR more than cutoff levels by number of all 90-day dead patients who received each NLR evaluation. ^#^: Specificity of day-1, day-4, and day-8 NLRs was calculated by dividing number of alive patients on day 91 with each NLR less than cutoff levels by number of all 91-day survived patients received each NLR evaluation.

**Table 6:** Prognostic factors in patients with AE-IPF (*n* = 86): Cox proportional hazard regression analysis; 90-day survival *.

Parameter	HR	95% CI	*p*-Value
**Univariate**			
**Before AE**			
Age, years	0.987	0.953–1.022	0.472
Sex, male/female	1.241	0.585–2.631	0.573
Smoking, CS or Ex/NS	1.478	0.722–3.027	0.285
Autoantibody, yes/no	0.993	0.485–2.033	0.986
PSL before AE, yes/no	1.571	0.884–2.795	0.124
LTOT, yes/no	1.562	0.898–2.719	0.114
**At the onset of AE (day 1)**			
Triggered, yes/no	0.776	0.332–1.813	0.558
PaO_2_/FiO_2_ ratio, Torr	0.997	0.994–1.001	0.130
PaO_2_/FiO_2_ ratio, ≤200/>200	1.956	1.075–3.556	0.028
KL-6, ×100 U/mL (*n* = 82)	1.006	0.983–1.030	0.590
WBC, ×10^8^/L	1.001	0.994–1.008	0.710
Neutrophils, ×10^8^/L	1.004	0.997–1.010	0.292
Lymphocytes, ×10^8^/L	0.921	0.875–0.968	0.001
NLR	1.034	1.016–1.053	<0.001
NLR >12.13/≤12.13	3.075	1.712–5.521	<0.001
CRP, mg/dL	1.001	0.971–1.033	0.933
LDH, U/L	1.002	0.999–1.004	0.216
HRCT, diffuse/non-diffuse	1.858	1.068–3.233	0.028
Multivariate *			
**Model 1**			
PaO_2_/FiO_2_ ratio, ≤200/>200	2.226	1.195–4.147	0.012
NLR	1.041	1.021–1.061	<0.001
**Model 2**			
PaO_2_/FiO_2_ ratio, ≤200/>200	2.387	1.290–4.417	0.006
NLR >12.13/≤12.13	2.906	1.635–5.166	<0.001

Abbreviations: AE, acute exacerbation; CI, confidence interval; CRP, C-reactive protein; CS, current smoker; EX, ex-smoker; FiO_2_, fraction of inspire oxygen; HR, hazard ratio; HRCT, high-resolution computed tomography; LDH, lactate dehydrogenase; LTOT, long-term oxygen therapy; NLR, neutrophil-to-lymphocyte ratio; NS, non-smoker; PaO_2_, arterial oxygen tension. *: Multivariate Cox proportional hazard regression analysis with stepwise selection procedure using parameters before AE and at the onset of AE. For Model 2, NLR (>12.13/≤12.13) was used instead of NLR.

**Table 7:** Prognostic factors in survival patients with AE-IPF on day 4 and day 8: Cox proportional hazard regression analysis; 90-day survival.

Parameter	HR	95% CI	*p*-Value
On day 4 (*n* = 79 *)			
**Univariate analysis**			
Oxygenation deterioration on day 4, yes/no	4.393	2.428–7.948	<0.001
Day-4 NLR	1.026	1.011–1.041	<0.001
Day-4 NLR, >14.90/≤14.90	3.075	1.712–5.521	<0.001
Neutrophils on day 4, ×10^8^/L	1.003	0.997–1.010	0.286
Lymphocytes on day 4, ×10^8^/L	0.924	0.870–0.981	0.009
LDH on day 4, U/L	1.003	1.001–1.005	<0.001
CRP on day 4, mg/dL	1.012	0.965–1.061	0.628
Multivariate analysis **			
**Model 1**			
Oxygenation deterioration on day 4, yes/no	3.949	2.138–7.293	<0.001
Day-4 NLR	1.024	1.009–1.040	0.002
LDH on day 4, U/L	1.003	1.001–1.005	0.008
**Model 2**			
Oxygenation deterioration on day 4, yes/no	3.553	1.920–6574	<0.001
Day-4 NLR, >14.90/≤14.90	3.395	1.834–6.282	<0.001
LDH on day 4, U/L	1.004	1002–1.006	0.008
**On day 8 (*n* = 71 ^§^)**			
**Univariate analysis**			
Oxygenation deterioration on day 8, yes/no	4.131	2.165–7.883	<0.001
Day-8 NLR	1.065	1.035–1.095	<0.001
Day-8 NLR, >10.56/≤10.56	5.451	2.784–10.671	<0.001
Neutrophils on day 8, ×10^8^/L	1.010	1.001–1.019	0.022
Lymphocytes on day 8, ×10^8^/L	0.927	0.889–0.968	<0.001
LDH on day 8, U/L	1.002	1.001–1.004	0.012
CRP on day 8, mg/dL	1.079	1.022–1.139	0.006
**Multivariate ^#^**			
**Model 3**			
Oxygenation deterioration on day 8, yes/no	2.849	1.366–5.942	0.005
Day-8 NLR	1.046	1.014–1.080	0.005
**Model 4**			
Oxygenation deterioration on day 8, yes/no	2.318	1.105–4.863	0.026
Day-8 NLR, >10.56/≤10.56	3.927	1.685–8.267	<0.001

Abbreviations: AE, acute exacerbation; CI, confidence interval; CRP, C-reactive protein; FiO_2_, fraction of inspire oxygen; HR, hazard ratio; LDH, lactate dehydrogenase; NLR, neutrophil-to-lymphocyte ratio; PaO_2_, arterial oxygen tension. *: Day-4 NLR was not evaluated in 1 patient. **: Multivariate Cox proportional hazard regression analysis with stepwise selection procedure using all parameters used for univariate analysis. Day-4 NLR and day-4 NLR (>14.90/≤14.90) were used for model 1 and model 2, respectively. ^§^: Day-8 NLR was not evaluated in 2 patients. ^#^: Multivariate Cox proportional hazard regression analysis with stepwise selection procedure using all parameters used for univariate analysis. Day-8 NLR and day-8 NLR (>10.56/≤10.56) were used for model 3 and model 4, respectively.

**Table 8:** Treatment for non-deteriorated cases according to day 4 and day 8.

Parameters	Higher NLR *	Lower NLR *	*p*-Value
**Day 4**	*n* = 21	*n* = 33	
Initial dose of PSL, mg/kg	0.909 (0.831–1.015)	0.930 (0.565–0.987)	0.950
Immunosuppressant, yes/no	8/13	21/12	0.095
CPA pulse, yes/no	3/18	5/28	1.000
AZP/CyA	3/3	9/8	1.000
Days from AE to IMs onset	7.5 (3.25–29.75)	15.0 (2.5–45.5)	0.756
Days from AE to IMs onset, ≤3/3<	2/6	7/14	1.000
Recombinant soluble TM, yes/no	2/19	4/29	1.000
PMX-DHP therapy, yes/no	3/18	9/24	0.329
Pirfenidone after AE, yes/no	0/21	2/31	0.516
**Day 8**	*n* = 10	*n* = 42	
Initial dose of PSL, mg/kg	0.932 (0.802–1.111)	0.918 (0.555–0.984)	0.318
IMs, yes/no	3/7	24/18	0.167
CPA pulse, yes/no	2/8	4/38	0.324
AZP/CyA	1/1	11/11	1.000
Days from AE to IMs onset	7 (3–14)	12.5 (1.25–41.75)	0.546
Days from AE to IMs onset, ≤7/7<	2/1	10/14	0.569
Recombinant soluble TM, yes/no	0/10	5/37	0.569
PMX-DHP therapy, yes/no	3/7	7/35	0.382
Pirfenidone after AE, yes/no	0/10	2/40	1.000

Abbreviations: AE, acute exacerbation; AZP, azathioprine; CPA, cyclophosphamide; IMs, immunosuppressants; NLR, neutrophil-to-lymphocyte ratio; PMX-DHP therapy, hemoperfusion therapy with polymyxin B-immobilized fiber column; PSL, prednisolone; TM, thrombomodulin. Number or median (interquartile range) of each parameter was shown. Each parameter was compared between higher NLR group and lower NLR group with Fisher exact test or Wilcoxon rank-sum test. *: Cutoff of day-4 and day-8 NLRs to determine higher and lower NLR was 14.90 and 10.56, respectively.

## Data Availability

We will share the data of this study upon reasonable request to the corresponding author; however, additional ethical institutional approval will be required. In addition, we might decline the request if it competes with our future research plan.
